# Congenital Heart Defects Are Rarely Caused by Mutations in Cardiac and Smooth Muscle Actin Genes

**DOI:** 10.1155/2015/127807

**Published:** 2015-03-10

**Authors:** Tatiana Khodyuchenko, Anna Zlotina, Tatiana Pervunina, Dmitry Zverev, Anna Malashicheva, Anna Kostareva

**Affiliations:** ^1^Institute of Molecular Biology and Genetics, Federal Almazov Medical Research Centre, 2 Akkuratova Street, Saint-Petersburg 197341, Russia; ^2^Endovascular Surgery Department, Federal Almazov Medical Research Centre, 2 Akkuratova Street, Saint-Petersburg 197341, Russia; ^3^St. Petersburg State University, Universitetskaya Nab. 7/9, St. Petersburg 199034, Russia; ^4^Department of Women and Child Health, Karolinska Institute, and Centre for Molecular Medicine, 17176 Stockholm, Sweden

## Abstract

*Background*. Congenital heart defects (CHDs) often have genetic background due to missense mutations in cardiomyocyte-specific genes. For example, cardiac actin was shown to be involved in pathogenesis of cardiac septum defects and smooth muscle actin in pathogenesis of aortic aneurysm in combination with patent ductus arteriosus (PDA). In the present study, we further searched for mutations in human *α*-cardiac actin (*ACTC1*) and smooth muscle *α*-actin (*ACTA2*) genes as a possible cause of atrial septum defect type II (ASDII) and PDA. *Findings*. Total genomic DNA was extracted from peripheral blood of 86 individuals with ASDs and 100 individuals with PDA. Coding exons and flanking intron regions of *ACTC1* (NM_005159.4) and *ACTA2* (NM_001613) were amplified by PCR with specific primers designed according to the corresponding gene reference sequences. PCR fragments were directly sequenced and analyzed. Sequence analysis of *ACTC1* and *ACTA2* did not identify any nucleotide changes that altered the coding sense of the genes. In *ACTC1* gene, we were able to detect one previously described nucleotide polymorphism (rs2307493) resulting in a synonymous substitution. The frequency of this SNP was similar in the study and control group, thus excluding it from the possible disease-associated variants. *Conclusions*. Our results confirmed that the mutations in *ACTC1* gene are rare (at least <1%) cause of ASDII. Mutations in *ACTA2* gene were not detected in patients with PDA, thus being excluded from the list of frequent PDA-associated genetic defects.

## 1. Introduction

Congenital heart disorders (CHDs) arise constantly all over the world with approximate frequency about 1%, slightly deviating depending on the geographic area. Despite the major progress in surgical treatment and rehabilitation of patients with CHDs, the medical, economic, and social burden from these disorders remains substantial. Etiologically, CHDs are associated with unfavorable environmental and embryotoxic factors. Apart from that, the genetic causes of CHDs are well established and include numerical and structural chromosomal aberrations, such as trisomy of 21 chromosome (Down syndrome) and chromosomal microdeletions such as DiGeorge syndrome [1]. The genetic etiology of CHDs was extensively studied during the last decade, leading to the description of mosaic chromosomal aberrations as well as single gene mutations causing the CHDs [2, 3]. The latter cases mostly include germ line and somatic mutations in cardiac transcription factors, such as* NKX2-5*,* GATA4*,* TBXx5*,* TBX20*, and* Notch1* [4–6]. Mutations in genes encoding structural and contractile cardiomyocyte proteins are associated with CHDs along with transcription factors. Thus, mutations in* MYH7*,* MYH6,* and* ACTC1* genes, encoding cardiac myosin heavy chain and cardiac actin, major contractile proteins of cardiomyocytes, can be found in patients with atrial and ventricular septum defects [7–10]. Originally, the mutations in these genes were described as a cause of cardiomyopathies—severe disorders with myocardial remodeling and altered cardiac function without inborn structural heart defects. The identification of sarcomeric gene mutations as a cause of familial CHDs underlined the role of these proteins in cardiac development. Up to now, only two studies have been published regarding the frequency of* ACTC1* mutations in patients with atrial septum defect type II (ASDII) [11, 12]. Therefore, extending knowledge on the frequency of cardiac actin mutation as a cause of ASD is of current importance.

Much less is known about genetic causes of another common CHD, namely, patent ductus arteriosus (PDA). Apart from mutations in cardiac transcriptional factors (such as* GATA6* and* TFAP2B*), mutations in genes encoding structural and contractile proteins of vascular smooth muscle cells were described in association with PDA [13, 14]. In latter case, mutations in smooth muscle actin (*ACTA2*) and smooth muscle myosin (*MYH11*) cause PDA associated with aortic aneurysm [15–17]. It is still unknown, however, whether* ACTA2* gene mutations can be associated with isolated cases of PDA without aortic aneurysm. The aim of our study was to assess the frequency of* ACTC1* mutations in patients with sporadic cases of ASDII and the frequency of* ACTA2* mutation in patients with isolated PDA without aortic aneurysm.

## 2. Methods

The study included 100 individuals with PDA and 86 individuals with ASDIIs who underwent cardiac endovascular interventions in Federal Almazov Medical Research Centre during 2010–2012. Family history of CHD was reported in 7% of PDA cases and in 6% of ASDII cases and included mainly concordant types of CHD and no cardiomyopathy cases. Male/female ratio was 53%/47% in both study groups; mean age at surgery was 5.8 in ASD group and 3.5 in PDA group. Syndromic patients, patients with cardiomyopathies or patients with other developmental anomalies, were not included in the study. Control DNA was obtained from 100 healthy individuals with no structural cardiac abnormalities confirmed by echocardiography.

The study was performed according to Helsinki Declaration and approval was obtained from the local ethics committees in St. Petersburg. Written informed consent was obtained from all subjects and their representatives prior to investigation. Total genomic DNA was extracted from peripheral blood using FlexiGene DNA Kit (Qiagen) according to the manufacturer's protocol. Coding exons and flanking intron regions of* ACTC1* (NM_005159.4) and* ACTA2* (NM_001613) were amplified by PCR with specific primers designed according to the corresponding gene reference sequences. Primer design is available upon request. PCR fragments were bidirectionally sequenced using a BigDye Terminator v3.1 sequencing kit and a 3130 Genetic Analyzer (Applied Biosystems). Sequencing results were analyzed with BioEdit 7.1 software and Sequencing Analysis 5.3.1 software (Applied Biosystems).

## 3. Results and Discussion

Sequence analysis of* ACTA2* and* ACTC1* did not identify any nucleotide changes that altered the coding sense of the genes or were not previously described as allelic variants. Analysis of* ACTC1* gene revealed one previously described nucleotide polymorphism rs2307493 in heterozygous state in one patient with ASDII, resulting in a synonymous substitution P309P (corresponding frequency 1.2%). Study of the control population revealed the same substitution in 2 out of 100 cases (corresponding frequency 2%). This genetic variant also exists in 1000 genomes and EVS databases with overall minor allele frequency 2.86% and minor allele frequency in Caucasian populations 1%, thus excluding it from the possible disease-associated variants.

The possible association between congenital heart disorders and mutations in cardiac and vascular smooth muscle actin genes has been shown earlier in several reports [7, 8, 15]. However, the frequency of these events was reported to be quite rare. Assessing a group of 31 CHD patients, Posch et al. reported that mutations in* ACTC1* gene encoding cardiac actin represented a rare cause of familial ASDII [12]. Similar results were yielded by Matsson et al., who assessed a group of 80 sporadic ASDII cases [11]. Our own study confirmed previously reported results, bringing additional evidence that* ACTC1* mutations are not common cause of atrium septum defects and contribute to at least ≤1% cases. We also showed that mutations in* ACTA2* gene encoding smooth muscle actin are not a common cause of sporadic cases of PDA, since we were not able to detect* ACTA2* mutations in a series of 100 PDA cases. Therefore, our study is the first bringing the evidence that mutations in* ACTA2* can be excluded from the list of frequent PDA-associated genetic defects.

## List of Abbreviations

CHD: Congenital heart defects
PDA: Patent ductus arteriosus
ASD: Atrial septal defects
MAF: Minor allele frequency.
